# Classification of comorbidity in trauma: agreement and reliability of the pre-injury ASA-PS Scale

**DOI:** 10.1186/1757-7241-20-S1-P3

**Published:** 2012-03-22

**Authors:** Kjetil G Ringdal, Nils Oddvar Skaga, Petter Andreas Steen, Morten Hestnes, Petter Laake, J Mary Jones, Hans Morten Lossius

**Affiliations:** 1 Department of Research, Norwegian Air Ambulance Foundation, Drøbak, Norway; 2 Division of Critical Care, Oslo University Hospital, Ullevål, Oslo, Norway; 3 Institute of Clinical Medicine, Faculty of Medicine, University of Oslo, Norway; 4 Norwegian National Trauma Registry, Oslo University Hospital, Oslo, Norway; 5 Department of Anaesthesiology, Division of Critical Care, Oslo University Hospital, Ullevål, Oslo, Norway; 6 Prehospital Centre, Division of Critical Care, Oslo University Hospital, Ullevål, Oslo, Norway; 7 The Ullevål Trauma Registry, Department of Research and Development, Division of Critical Care, Oslo University Hospital, Ullevål, Oslo, Norway; 8 Department of Biostatistics, Institute of Basic Medical Sciences, Faculty of Medicine, University of Oslo, Norway; 9 Mathematics Department, School of Computing and Mathematics, Faculty of Natural Sciences, Keele University, Keele, UK; 10 Department of Surgical Sciences, Faculty of Medicine and Dentistry, University of Bergen, Norway

## Background

Pre-injury comorbidity influence outcome in severely injured patients. Pre-injury comorbidity graded according to the American Society of Anesthesiologists Physical Status (ASA-PS) classification system (Table 1) is an independent predictor of survival in trauma patients, and is suggested as a comorbidity score in the Utstein Trauma Template. Little is known about the levels of agreement and reliability of pre-injury ASA-PS scores. The objective of the study was to examine if pre-injury ASA-PS was a reliable scale for grading comorbidity of trauma patients among a representative group of trauma registry coders.

## Methods

18 Norwegian trauma registry coders were invited to participate in an agreement and reliability study, of which 50 real but anonymised patient medical records were distributed. Agreement was analysed using proportions, and reliability was analysed using quadratic weighted kappa (κ_w_) with 95% confidence intervals (CI) as the primary outcome measure, and unweighted kappa (κ) analysis that included ‘ Unknown’ values as secondary outcome measure.

## Results

15 of the invitees responded to the invitation, and ten participated. Agreement between the participants and the gold standard was 73.6%. After recoding the ‘ Unknown’ values into pre-injury ASA-PS 1, agreement was 80.4%. We found moderate (κ_w_=0.76, 95%CI 0.64-0.87) to substantial (κ_w_=0.95, 95%CI 0.89-0.99) rater-to-gold standard reliability using κ_w_ (Table 2 and Figure 1), and fair (κ=0.46, 95%CI 0.29-0.64) to substantial (κ=0.83, 95%CI 0.68-0.94) reliability using κ (Table 3 and Figure 2). Inter-rater reliability showed a mean κ_w_=0.82 (95%CI: 0.80-0.84), and a mean κ=0.57 (95%CI: 0.54-0.60).

## Conclusions

Rater-to-gold standard agreement and reliability varied from moderate to substantial for the primary outcome measure, and from fair to substantial for the secondary outcome measure. The findings of the study indicate that the pre-injury ASA-PS scale may be a reliable score for classifying comorbidity in trauma patients. The Utstein Template should specify how to handle unknown values.

